# Molecular Dynamics Simulations of Adsorption of SARS-CoV-2 Spike Protein on Polystyrene Surface

**DOI:** 10.1021/acs.jcim.2c00562

**Published:** 2022-08-04

**Authors:** Mehdi Sahihi, Jordi Faraudo

**Affiliations:** Institut de Ciencia de Materials de Barcelona (ICMAB-CSIC), Campus de la UAB, Bellaterra, E-08193 Barcelona, Spain

## Abstract

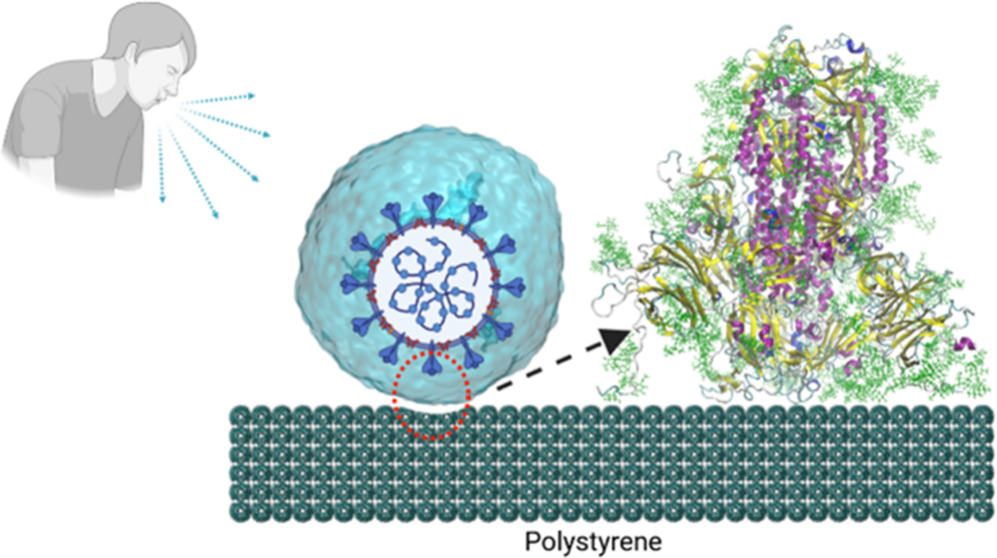

A prominent feature of coronaviruses is the presence of a large glycoprotein spike (S) protruding from the viral particle. The specific interactions of a material with S determine key aspects such as its possible role for indirect transmission or its suitability as a virucidal material. Here, we consider all-atom molecular dynamics simulations of the interaction between a polymer surface (polystyrene) and S in its up and down conformations. Polystyrene is a commonly used plastic found in electronics, toys, and many other common objects. Also, previous atomic force microscopy (AFM) experiments showed substantial adhesion of S over polystyrene, stronger than in other common materials. Our results show that the main driving forces for the adsorption of the S protein over polystyrene were hydrophobic and π–π interactions with S amino acids and glycans. The interaction was stronger for the case of S in the up conformation, which exposes one highly flexible receptor binding domain (RBD) that adjusts its conformation to interact with the polymer surface. In this case, the interaction has similar contributions from the RBD and glycans. In the case of S in the down conformation, the interaction with the polystyrene surface was weaker and it was dominated by glycans located near the RBD. We do not find significant structural changes in the conformation of S, a result which is in deep contrast to our previous results with another hydrophobic surface (graphite). Our results suggest that SARS-CoV-2 virions may adsorb strongly over plastic surfaces without significantly affecting their infectivity.

## 1 Introduction

Epidemic outbreaks of respiratory viral diseases represent a serious issue to public health, as demonstrated historically by influenza pandemics and now by the ongoing COVID-19 pandemic. The SARS-CoV-2 virus (responsible for the COVID-19 disease) is the third documented spillover of an animal coronavirus to humans in only two decades,^1^ and it has the highest transmission rate among them.^2^ The outbreak originated in December 2019 in Wuhan, China, and expanded so fast around the world that the World Health Organization (WHO) declared a Public Health Emergency of International Concern (PHEIC) on 30 January 2020, followed by a declaration of global pandemic on 11 March 2020.^3,4^ As of 13 April 2022, over 501.2 million people were infected. Generally, breaking the transmission chain in all of the viral diseases is a top priority to control them. Transmission is due to respiratory secretions or droplets expelled by infected individuals. These secretions not only may affect other persons (direct transmission), but they are also able to contaminate inanimate surfaces. Viable SARS-CoV-2 virus can remain active on surfaces for periods ranging from hours to days, depending on the ambient environment (including temperature and humidity) and the type of surface.^5^ Transmission also occurs indirectly through touching surfaces, textiles, or objects contaminated with the virus followed by touching the mouth, nose, or eyes. For this reason, generic antiviral disinfection measures (appropriate for previously known enveloped viruses) are recommended for hands, surfaces, and materials, e.g., applying alcoholic disinfectants or soaps containing surfactants. Design of more focused recommendations, more efficient disinfection strategies, and development of virucidal surfaces or textiles will be possible with a fundamental knowledge of the physicochemical aspects of the virus interaction with materials. Experimental evidence^6^ suggests that the interaction of the virus with surfaces of different materials is highly specific, but at the present time, the fundamental aspects of virus–material interactions are not known. The lack of fundamental, physicochemical knowledge of the virus–surface interaction is in contrast to the wealth of atomistic detailed information available about the virus and its molecular interactions. The structure of the SARS-CoV-2 virus is well known: it has the typical structure of a coronavirus with an envelope containing lipids and proteins, which protects the nucleocapsid that packages the viral RNA. The large protruding glycoprotein spikes on the envelope, typical of the Coronaviridae family of viruses, are responsible for the interaction with host cell receptors and the environment. The molecular structure with atomistic coordinates of the SARS-CoV-2 virus spike (S) protein was published as early as March 2020.^7−10^ Trajectories from atomistic molecular dynamics (MD) simulations are also available in specific repositories, such as the COVID-19 Molecular Structure and Therapeutics Hub (https://covid.bioexcel.eu/) (see a complete list in ref (11)). So far, these advanced atomistic simulation studies have focused on elucidating the molecular interactions for drug or vaccine development but have ignored questions related to disease propagation, such as virus interaction with materials.

Polymers are a category of advanced materials widely used in our daily life, e.g., personal protective equipment (PPE), clothes made from synthetic fibers, various dishes, cookware, fiberglass, plastic bags, paints, glues, artificial organs, etc.^12^ Hence, indirect transmission of viral diseases through touching contaminated polymeric surfaces or aggregation of viruses on the surface of polymeric materials should be considered with more attention. Recent experiments have shown that SARS-CoV-2 was more stable than SARS-CoV-1 on plastic compared to copper and cardboard, and the viable virus was detectable up to 72 h after application to the plastic surface.^5^ The resistance of positively charged polymers against different viruses has also been proved previously.^13^ Polystyrene, polyethylene, polyester, and polycarbonate are variants of polymers that could be used to produce medical face masks and other PPE. Among these polymers, polystyrene has also become one of the most commonly used plastics in many other aspects of our lives, e.g., in household appliances and toys, in furniture and electrical articles, in vehicles, in buildings, and in packaging.^14^ Therefore, studying the adsorption of viruses on the surface of polystyrene and finding the mechanism of their interaction have been of scientific interest since about 1980.^15^ Murray and Parks found that viruses weakly adsorb to organic surfaces like polystyrene.^15^ In another study, Al-Kaissi and Mostratos investigated the adsorption of influenza to polystyrene.^16^ Their results showed that two of the three studied types of influenza viruses were still active after adsorption to the surface of polystyrene. The adsorption of alfalfa mosaic virus (AMV) antigens to the polystyrene of enzyme-linked immunosorbent assay (ELISA) plates has also been investigated and it was shown that adsorption was a slow and temperature-dependent procedure.^17^ To characterize the interaction of SARS-CoV-2 virions with surfaces, Xie et al.^18^ employed atomic force microscopy (AFM) to measure the adhesion force and adhesion energy of the SARS-CoV-2 S protein with a series of inanimate surfaces including a large diversity of hydrophobic and hydrophilic materials such as glass, metals, fabrics, and plastics. Interestingly, polystyrene was found to show the strongest adhesion force for reasons that were not identified in the experiments. Hydrophobic interactions were suggested as a possible mechanism^18^ although this is not obvious, given the structure of the protein, which mainly exposes hydrophilic groups. Our main motivation for the present study is precisely to identify these mechanisms at the atomistic level. As we will see later here, our simulations show that these strong interactions are due to the glycans covalently attached to S and, depending on the conformation of the S protein, on the amino acids located near and at the receptor binding domain (RBD).

**Figure 1:**
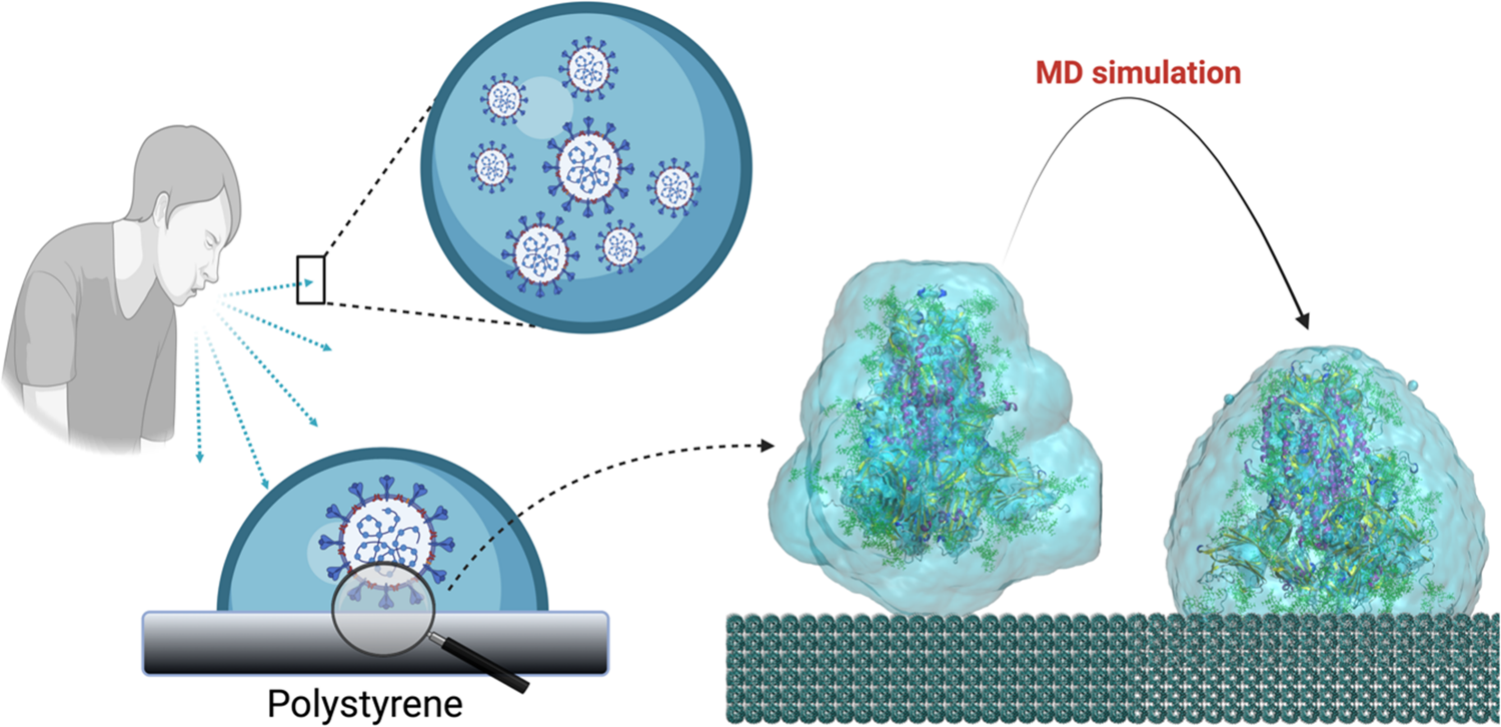
Cartoon illustrating the rationale behind the present study. The sneezing or coughing of an infected individual generates droplets that contain virions that may land over a surface (polystyrene in this example). The contact of the virion particle with the surface is made through their S proteins. The S1 subunit is the head of the protein that is exposed to the exterior and is responsible for contact with the polystyrene surface. In our simulation model, as a simplified representation of this process, we consider the S1 subunit of S protein inside a water droplet approaching a polystyrene surface. The adhesion between the S protein and the surface is then simulated using atomistic MD simulations. (Created with BioRender.com).

At this point, we recall that, in the case of SARS-CoV-2, the interaction of the virus with the environment takes place through its S protein (Figure 1). Therefore, the identification of the atomistic origin of the interaction mechanisms between the S protein and surfaces is not only relevant for the interpretation of experimental data regarding the S-protein alone but also is key to the understanding of the interaction between SARS-CoV-2 virions and surfaces.

In our simulations, we will consider the two different conformations (up and down) that have been resolved for the trimeric S glycoprotein based on the orientation of its RBD.^7,19^ The “up” conformation corresponds to an S protein with one of its three RBDs exposed (ready for binding to a receptor), and the “down” conformation corresponds to all three RBDs hidden. High-resolution experimental images of SARS-CoV-2 virions reveal that the proportion of up and down conformations of the S protein are approximately 1:1.^20^ Most of the published MD simulation studies have only investigated the interaction of the up conformation of S protein with the surface of the materials, and only a few studies have considered the interaction of down conformation, as well.^21^ Hence, in the present study, we investigate the interaction between up and down conformations of the S protein and a polystyrene slab using the all-atom MD simulation method. The specific objectives of this study are: (i) to clarify the molecular and atomic details of the interaction between SARS-CoV-2 virus and polystyrene (Figure 1); (ii) to reveal the difference between up and down conformations of the S protein for interaction with polystyrene surface; and (iii) to complement our previous works on the interaction between SARS-CoV-2 and different types of materials (hard, soft, hydrophobic, and hydrophilic surfaces).^22,23^

## 2 Simulation Methods

### 2.1 System Preparation

The simulated systems consisted of a water droplet containing a SARS-CoV-2 S protein (in up and down conformations) initially located near a polystyrene surface. The S protein consists of three identical polypeptide chains and is divided into the S1 (residues 1–1146 per chain) and S2 (residues 1146–1273 per chain) subunits. The S1 subunit is the head of the protein that is exposed to the exterior and is responsible for contact with the polystyrene surface. However, the S2 subunit links the protein to the virion. Fully glycosylated structures of the S1 subunit of SARS-CoV-2 S protein were taken from CHARMM-GUI archive^24^ (PDB IDs: 6VSB and 6VXX for up and down conformations, respectively; Figure S1). The downloaded structures are based on cryo-electron microscopy (cryo-EM)-resolved crystal structures, reported by Walls et al.,^19^ and have the predicted missing residues and the linked glycans reported by Woo et al.^25^ The binding glycans may affect the interaction of the S protein with the polystyrene surfaces. Each conformation consists of 165 glycans per subunit of the S protein. The obtained structures contain 72 990 atoms, and their total charge (at pH = 7) is −15 *e*. The S protein structures were solvated using “gmx editconf” and “gmx solvate” modules of gromacs^26−29^ in a cubic box, and then all of the water molecules beyond 3 Å of a solvation shell were removed using Visual Molecular Dynamics (VMD) software.^30^ The number of water molecules added to solvate the glycosylated S protein were 83 809 and 67 041 for up and down conformations, respectively. We also neutralized the systems by adding K and Cl ions at a concentration of 150 mM.

The Maestro-Schrodinger software was used (Schrödinger, LLC, New York, NY) to prepare the structure of a polystyrene slab with dimensions of 30.02 × 35.02 × 6.10 nm^3^ consisting of 222 polystyrene chains in six layers. Every single chain of polystyrene contains 150 monomers and single chains separated from each other by approximately 2 Å. The final density of the polystyrene is about 0.92 g cm^–3^, which is very close to its experimental value (≈1.05 g cm^–3^).^31^ The total number of atoms was 849 958 and 799 464 for systems composed of a polystyrene slab with a water droplet and a S protein inside in its up and down conformations, respectively. As a reference simulation, we have considered the wetting behavior of the polystyrene with a water droplet without any protein inside. For the wetting calculations, a water droplet with a diameter of about 0.75 nm (6845 water molecules) was generated using VMD software.^30^ The solvated and neutralized structures of the S protein (and also the water droplet for wetting calculation) were placed on top of the polystyrene slab, and their distance was set to approximately 5.0 Å (Figure 1).

### 2.2 MD Simulations

All of the MD simulations were done using the simulation package GROMACS version 2019.3^26−29^ for the S protein–polystyrene and water–polystyrene complexes. The CHARMM36 force field was employed in all of the simulations. This force field considers parametrization of carbohydrate derivatives, polysaccharides, and carbohydrate–protein interactions.^32^ The TIP3P water model included in CHARMM36 is also used in our simulations. The polystyrene chains were also parameterized using the same force field. The CHARMM36 atom types and their charges used for polystyrene are presented in Figure S2 and Table S1. This force field has been previously used and verified for MD simulation of polystyrene–bioactive ligand complexes.^33^ The systems were placed in the center of the cubic box, and the minimum distance between the system and the box boundaries was set to 1.0 nm. The lowest three layers of the polystyrene slab were geometrically frozen during the simulations to approximate a realistic polymeric slab configuration. Integration of the equations of motion was done at a time step of 2 fs with full periodic boundary conditions (PBC) applied along the three Cartesian directions. The systems were energy-minimized using the conjugate gradient (CG) method, with 1 × 10^–6^ (kJ mol^–1^ and kJ mol^–1^ nm^–1^ for energy difference and RMS force, respectively) convergence criteria. Then, we performed a 200 ns NVT production run (2 ns for wetting calculations) at 300 K using a Berendsen thermostat^34^ with a damping constant of 0.1 ps. This thermostat has been widely employed in previous simulation works of proteins, showing good agreement with experiments.^35−38^ During the simulations, a 1.0 nm cutoff for Lennard-Jones (LJ) and Coulomb interactions was applied and the particle mesh-Ewald method^39,40^ was used for long-range electrostatics. The LINCS method^41^ was also used as a constraint algorithm. All of the images were generated with VMD software.^31^

## 3 Results and Discussion

### 3.1 Wetting Behavior of the Polystyrene Surface

**Figure 2:**
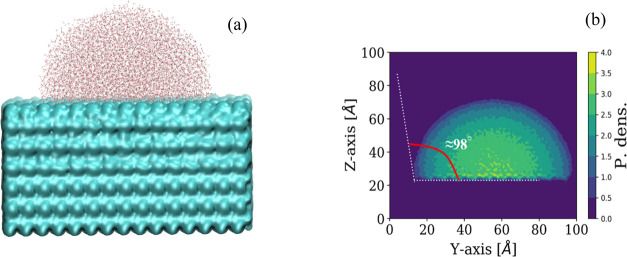
(a) Final configuration of water droplet and (b) its equilibrium contact angle on the surface of polystyrene surface.

To verify the accuracy of the used TIP3P water model and force field parameters of the polystyrene, we characterized the wetting behavior of the polystyrene by placing a water droplet on top of the polystyrene surface. As shown in Figure S3, the average RMSD value of the water droplet is about 3.99 ± 0.03 nm. In fact, the RMSD of the system reached equilibrium and fluctuated around its mean values after about 600 ps, indicating that the system well behaved thereafter and could be analyzed in its equilibrium state to calculate the equilibrium contact angle. Figure 2 shows the final configuration of the water droplet and its equilibrium contact angle on the surface of the polystyrene slab. The analysis of the results showed a contact angle of about 98°, i.e., we more or less recovered the water drop equilibrium contact angle value (∼90–98°) that has experimentally been observed for polystyrene.^42^ Although the surface tension of the water TIP3P model is lower than the experimental value of the water–air interface,^43^ it is remarkable that our simulation results, for wetting corresponding to wetting by a nanoscopic water droplet, are in good agreement with the macroscopic contact angles measured using multiple-interval reflection infrared (MIR-IR) dichroism. Hence, it could be concluded that the use of the TIP3P water model in combination with the force field parameters for polystyrene employed here is in agreement with experiments and they can be used in our simulation of the adsorption of hydrated S protein onto the polystyrene surface.

### 3.2 Adsorption of S Protein onto the Surface of Polystyrene

**Figure 3:**
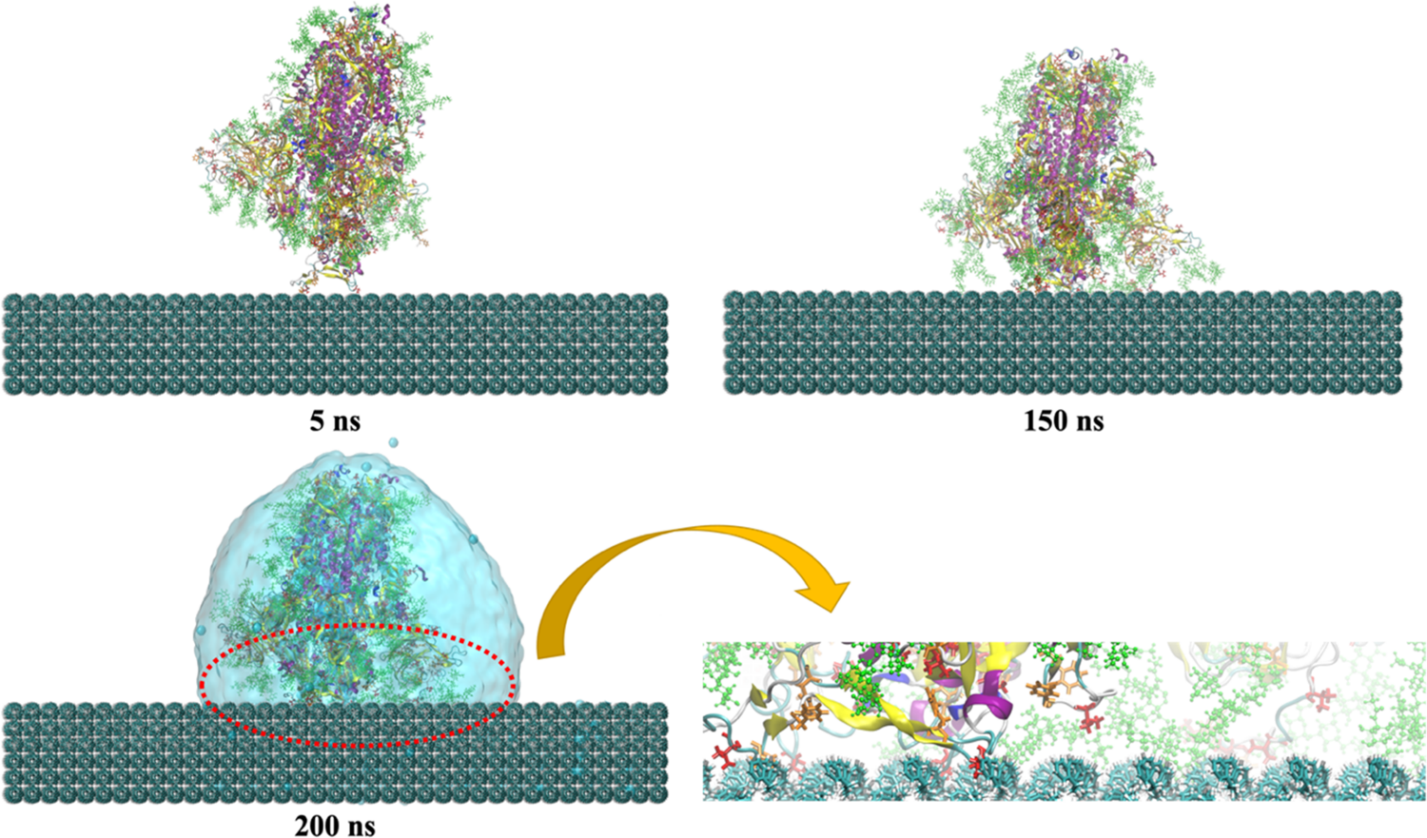
Representative snapshots (at different times) of the MD simulation of the up conformation of the S protein adsorbed onto the surface of polystyrene. For the sake of simplicity, we show the water droplet embedding the S protein only for the final snapshot (200 ns). We also show a zoom of the contact between S and the surface, emphasizing the groups involved in the interaction. Val and Phe amino acids are shown in bond representation (in red and orange colors, respectively). Glycans are shown in green in CPK representation.

Trajectories for the up and down conformations show that each conformation undergoes a different process during adsorption on the surface of polystyrene. For the up conformation, the interaction starts at about 5 ns by the Val and Phe amino acid residues in the RBD. Afterward, at about 14 ns, in addition to more Val and Phe residues in the N-terminal of the protein, some of the glycan groups were able to interact with the surface of the polymer and stabilize the system. Then, the protein adjusted its spatial conformation again with more contacts to the surface and achieved the relatively stable intermediate state at about 40 ns. More amino acid residues and glycan groups of the protein are adsorbed onto the surface of polystyrene as time evolves, and finally, the protein achieves its relatively stable state at about 150 ns and remains stable until the end of simulation time. Figure 3 represents that Val and Phe residues of up conformation RBD have major roles in contact initiation to polystyrene. Hence, the main driving forces for adsorption are hydrophobic and π–π interactions of polystyrene with Val and Phe residues, respectively.

**Figure 4:**
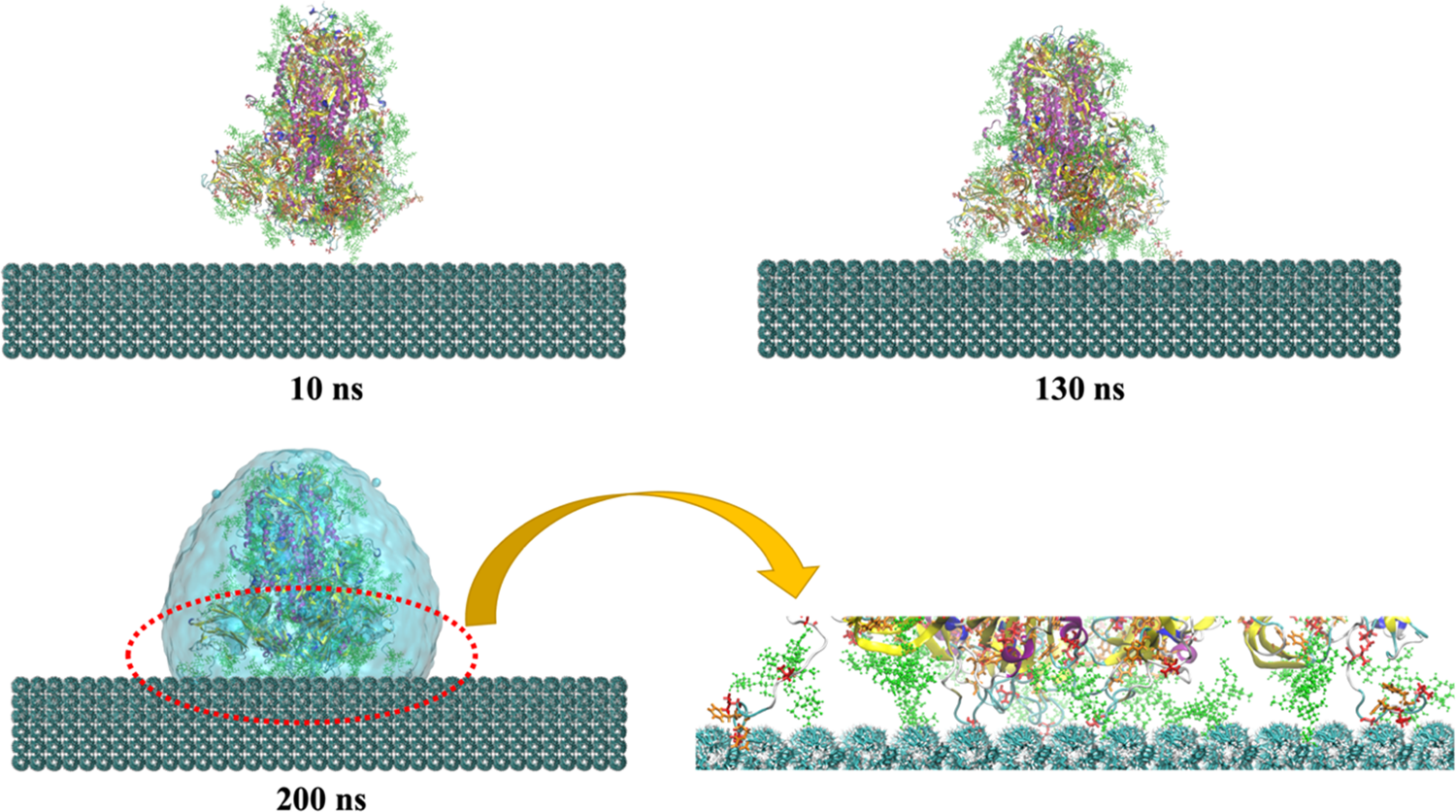
Representative snapshots (at different times) of the MD simulation of the down conformation of the S protein adsorbed onto the surface of polystyrene. For the sake of simplicity, we show the water droplet embedding the S protein only for the final snapshot (200 ns). We also show a zoom of the contact between S and the surface, emphasizing the groups involved in the interaction. Val and Phe amino acids are shown in bond representation (in red and orange colors, respectively). Glycans are shown in green in CPK representation.

For the down conformation, the interaction starts later than for the up conformation (at about 10 ns) due to the lack of open RBD in its structure. Figure 4 shows that the contacts between glycan groups and the surface of the polymer are the main driving force for the adsorption. These contacts decrease the distance of the protein and the polymer and then, at about 20 ns, in addition to more glycans, some Val and Phe residues of the protein would be accessible to interact with the surface of the polymer and stabilize the system at its intermediate state until about 130 ns. Then, the protein adjusted its spatial conformation and achieved a relatively stable state until the end of the simulation. The hydrophobic nature of the polystyrene due to the presence of styrene aromatic rings makes it favorable for hydrophobic and aromatic amino acid residues to be adsorbed.

**Figure 5:**
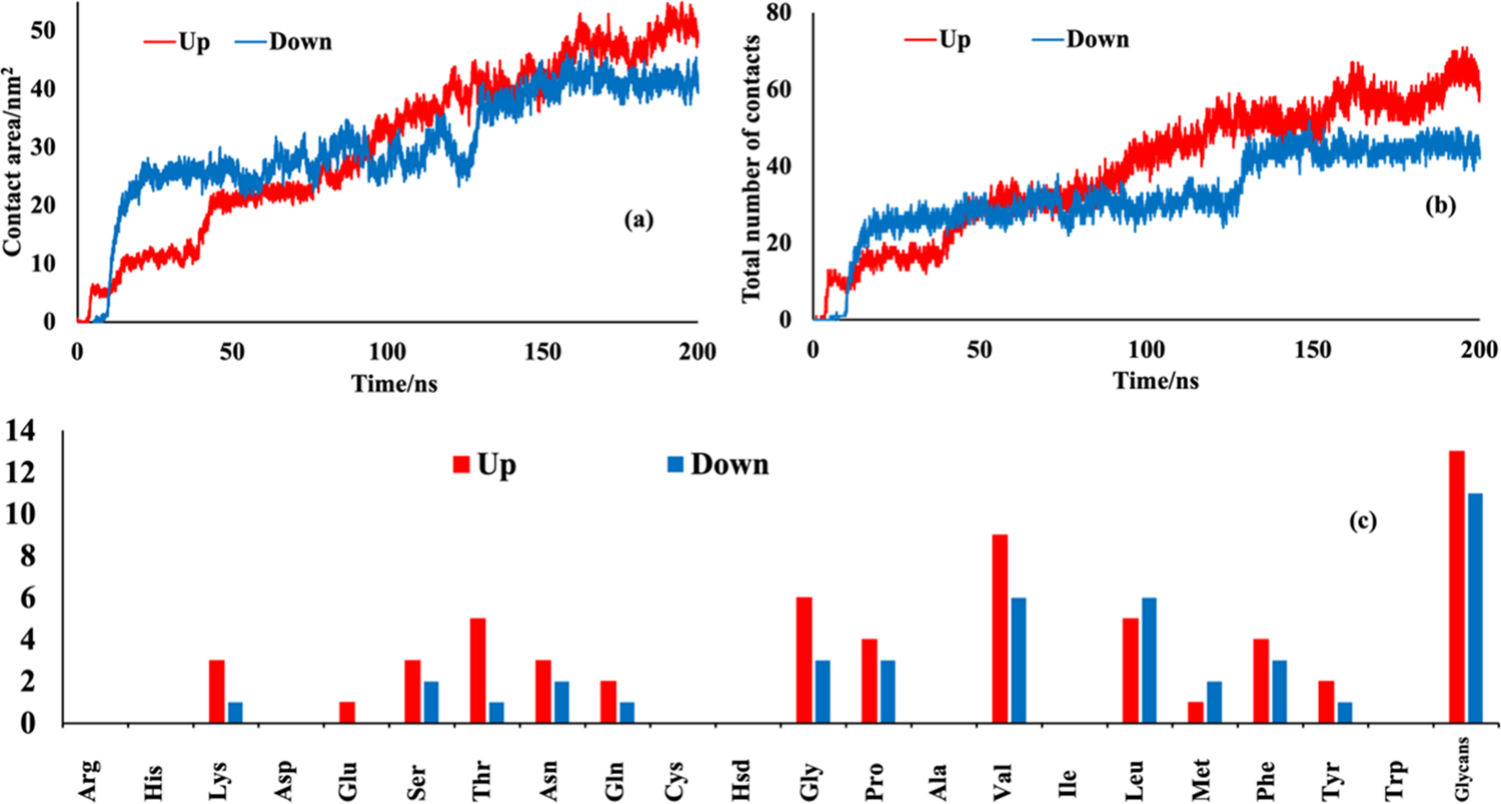
(a) Contact area between the S protein and polystyrene surface as a function of time; (b) total number of S protein residues in contact with polystyrene surface as a function of time; and (c) average number of S amino acids (three-letter code) in contact with polystyrene surface at equilibrium.

The contact area (Figure 5a) is calculated from the solvent accessible surface area as in ref (44). A TCL script for VMD, implementing a maximum distance of 0.35 nm as a cutoff value, was used to count the total number of amino acid residues in contact with polystyrene at each time step.^23^ The total number of the up conformation residues in contact with the polystyrene surface (Figure 5b) is larger than for the case of down conformation. This observation could be related to the accessibility of the exposed RBD of S in the up conformation. The exposure of this protruding RBD causes a contact of S with the surface of the polymer that starts earlier than in the case of S in the down conformation (hidden RBD). The changes in contact area and total number of contacts are completely in agreement with the adsorption mechanism stated and presented in Figures 3 and 4. Figure 5c represents the number of S amino acid residues (in the final time frame of the trajectories) in contact with polystyrene, classified by the type of amino acid. As expected (see Figures 3 and 4), the most abundant residue of up conformation in contact with the polystyrene surface is Val, which corresponds to hydrophobic interactions. However, Phe residues, which correspond to π–π interactions with styrene rings, can be considered as another important residue in stabilizing the protein on the surface of polystyrene. Also, there is a considerable number of contacts with other hydrophobic (Leu, Pro, and Gly) and aromatic (Tyr) amino acid residues. For the down conformation of S protein, glycans and Val amino acid residues have the highest number of contacts (11 and 6 contacts, respectively) with the surface of the polymer. However, there are six more contacts with Lue residues that are classified as hydrophobic amino acids, as well.

**Figure 6:**
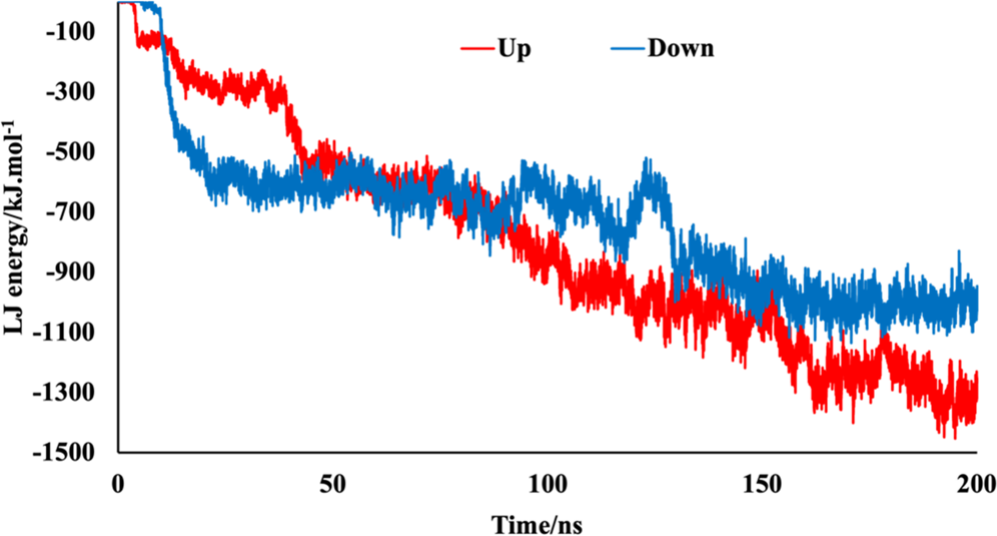
Lennard-Jones interaction energy between the S protein (in the up and down conformations) and the polystyrene surface as a function of time calculated from our MD simulations.

Figure 6 represents the trend of interaction energy with the evolution of time that is totally consistent with the contact details discussed before. When the S protein in the up conformation was adsorbed onto the polystyrene surface, the interaction energy and contact area changed rapidly at about 5 ns (LJ energy decreased and contact area increased). Other considerable changes were observed at about 40 and 150 ns and remained stable at about −1267.22 ± 61.86 kJ mol^–1^ and 49.21 ± 2.32 nm^2^ finally, for LJ interaction energy and contact area, respectively. However, when the down conformation adsorbed onto the polymer surface, the interaction energy and contact area changed (LJ energy decreased and contact area increased) between 10 and 130 ns and remain almost constant at about −946.26 ± 45.16 kJ mol^–1^ and 39.38 ± 2.05 nm^2^ until the end of the trajectory. Hence, not only the mechanisms of contact to the polystyrene surface are different for the up and down conformation but also their interaction energies are different (a more than 25% difference).The difference in the interaction energy is due to the different area of contact for each conformation. If we compute interaction energy per unit contact area, we have for the up case ≈26 kJ mol^–1^ nm^–2^ ≈ 43 mJ m^–2^ and ≈24 kJ mol^–1^ nm^–2^ ≈ 40 mJ m^–2^ for the down case. Therefore, the affinity of the S protein for polystyrene is larger in the up conformation compared with that of the down conformation. It is also interesting to compare the obtained interaction energy with the adhesion energies estimated from adhesion forces in AFM experiments.^18^ In these experiments, the forces between a tip covered by S proteins and a polystyrene surface were converted to adhesion energies using the Derjaguin–Muller–Toporov model, obtaining a surface energy of 11 mJ m^–2^ between the tip and a polystyrene surface. We do not know the density of S proteins over the tip, but according to Soloviev et al.,^45^ the maximum surface coverage of S proteins can be expected to be of ≈32%. Using this estimate, we infer from the AFM experiments that the adhesion energy of a single S should be of the order of 11/0.32 ≈ 34 mJ m^–2^ in agreement with our MD estimates.

Further insight into the origin of the S-surface interaction can be obtained by decomposing the total interaction energy into the interaction energies of the different parts of the S protein with the surface of the polystyrene slab (Table 1). The results indicated that RBD and glycan parts of the up conformation show the highest interaction energy. Also, glycan groups are the main responsible part for the interaction of polystyrene and the S down conformation. These observations are absolutely in agreement with the adsorption mechanisms mentioned above.

**Table 1:** Decomposition of the Interaction Energy between S Protein of SARS-CoV-2 (Up and Down Conformations) and Polystyrene Surface and the Different Parts of the Protein

	protein	RBD	glycans	total	
Interaction energy/kJ mol^–1^	–289.13 ± 14.53	–453.29 ± 23.07	–524.80 ± 26.37	–1267.22 ± 61.86	up
	–260.86 ± 13.09	–183.58 ± 10.83	–501.82 ± 26.15	–946.26 ± 45.16	down

### 3.3 Conformational Change of the S Protein

**Figure 7:**
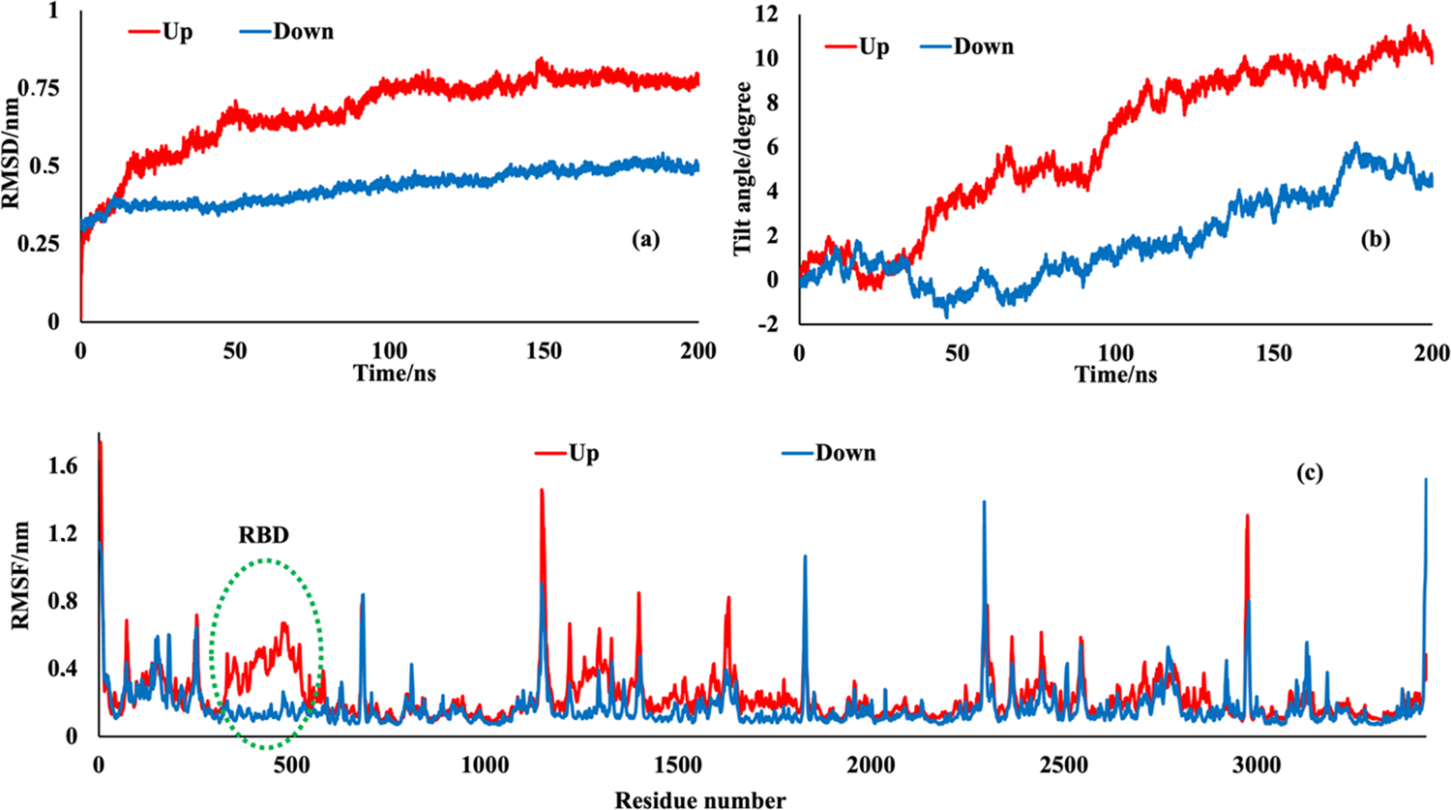
(a) RMSD of all backbone carbon atoms of S protein during 200 ns MD simulations; (b) tilt angle of the S protein with respect to the polystyrene surface as a function of time; and (c) root-mean-square fluctuation (RMSF) of S protein amino acid residues for MD simulation of the up and down conformations.

To understand the conformational changes of the up and down S proteins, we calculated their root-mean-square deviation (RMSD) relative to the crystal structure and without hydrogen atoms and glycans. As shown in Figure 7a, the average RMSD values of S protein were about 7.62 ± 0.31 and 4.96 ± 0.19 Å for up and down conformations, respectively. Hence, it can be concluded that the structural change of the up conformation adsorbed onto the surface of polystyrene is more than the down conformation. Furthermore, analysis of Figure 7a shows that the RMSD of the systems reached equilibrium and fluctuated around their mean values after about 150 and 130 ns (for up and down conformations, respectively), indicating that these systems well behaved thereafter.

Figure 7b represents the tilt angle of the protein with respect to the polystyrene surface. The tilt angle between the S protein and the *z*-axis (the axis perpendicular to the surface) was computed using “gmx bundle” module of the gromacs. The final equilibrium angles with the *z*-axis are about 10.1 and 4.2° for the up and down conformations, respectively. In both cases, the protein deviates only slightly from its original orientation (following the *z* axis and perpendicular to the surface), but the deviation is larger for the up conformation (compare also Figures 3 and 4). This observation is consistent with the above-mentioned results that show the higher interaction energy and contact area of the up conformation.

The residue-based root-mean-square fluctuation (RMSF) is calculated based on average positions of amino acids to evaluate their local dynamical variation and identify the regions of the protein that have high structural changes and fluctuations during the simulation. As shown in Figure 7c, the amino acid residues located in the N- and C-terminals of the S protein monomers have the highest RMSF due to their inherent high flexibility. Furthermore, the RMSF values for most of the residues in the up conformation are higher than the RMSF values in the down conformation. It means that the combination of up conformation of the S protein with polystyrene, which shows a higher interaction energy, makes the up conformation more flexible than down conformation. This observation is similar to the previous study about the interaction of M_pro_ of SARS-CoV-2 with graphene oxide, defective graphene, and intact graphene.^46^ Hence, it can be stated that, interestingly, the amino acid residues located at RBD with up conformation (Arg319-Phe541) have an obvious higher RMSF than similar amino acid residues in down conformation. It means that these highly flexible amino acid residues can adjust their conformation to start the interaction with the polymer surface.

**Figure 8:**
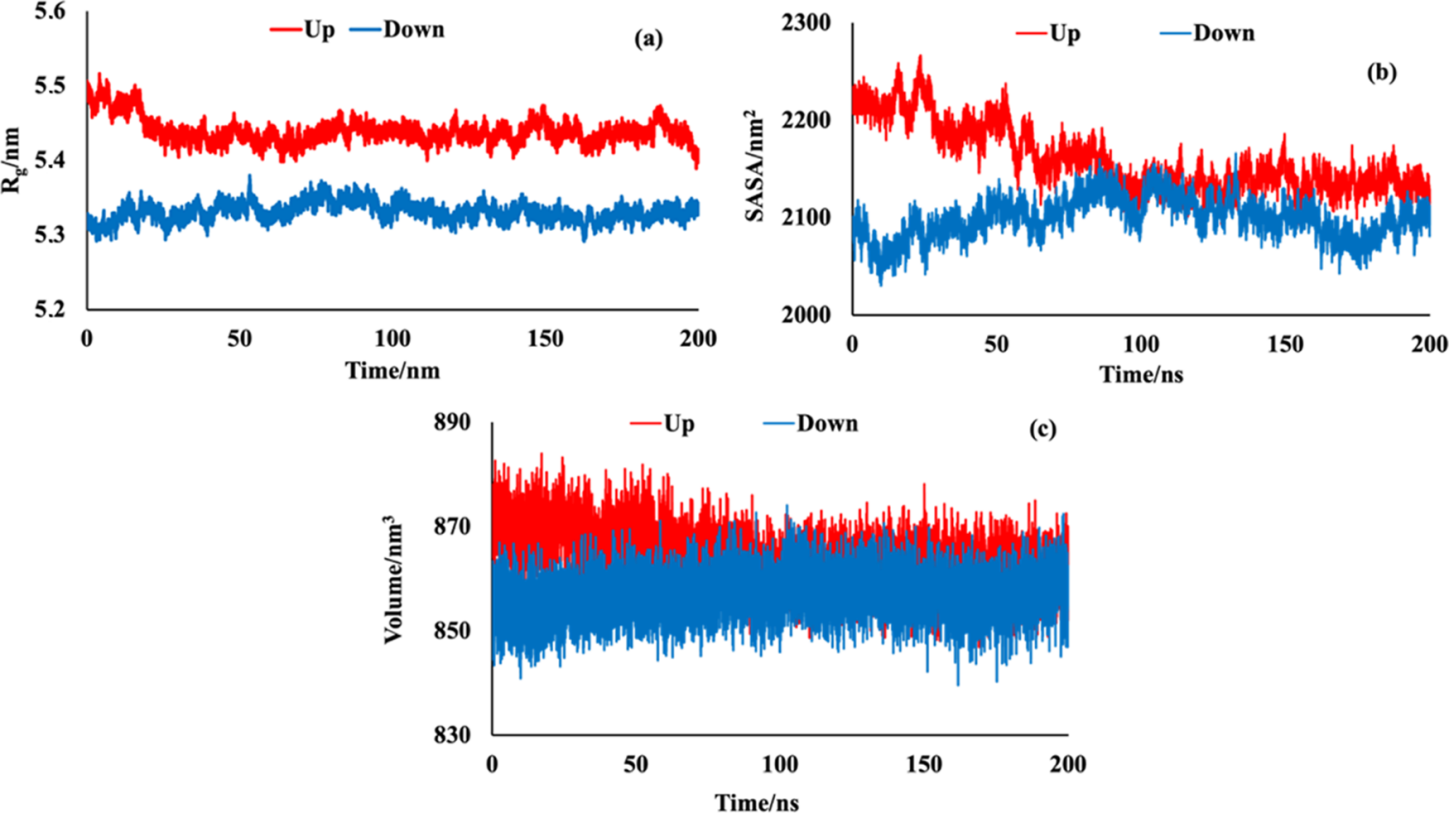
Time evolution of (a) *R*_g_, (b) SASA, and (c) volume of SARS-CoV-2 S protein during its interaction with polystyrene surface.

Radius of gyration (*R*_g_), solvent accessible surface area (SASA), and protein volume can be considered as indices of compactness, stability, and folding state of a protein. As can be seen in Figure 8a, the initial *R*_g_ value of the up conformation (glycosylated protein) is higher than that of the down conformation (5.48 and 5.32 nm, respectively). However, during the 200 ns of MD simulation trajectory, their *R*_g_ value of the up conformation decreases and that of the down conformation increases. Indeed, they show different conformational change pathways as it was expected due to their different adsorption mechanisms. However, their final folding states are almost the same as we anticipated from their interaction energy and contact area profiles (Figures 5 and 6). In these systems, the *R*_g_ values were stabilized at about 130–150 ns, indicating that the MD simulation achieved equilibrium thereafter. Figure 8b,c represents the changes in the SASA and S protein volume during the interaction of SARS-CoV-2 S protein (up and down conformations) with the polystyrene surface. Both of the properties show the same behavior as *R*_g_, interaction energy, and contact area.

Finally, the secondary structure of the S protein was analyzed using the DSSP module.^47^ The result provides the α-helix, β-sheet along with the other secondary structure contents of the S protein. It is easy to note that the main secondary structures of the protein in the presence of the polystyrene slab maintain rather stable for both the up and down conformations during the whole MD simulation time (Figure S4). Therefore, during the interaction of the protein with the polystyrene surface, the tertiary structures of protein up and down conformations have been changed and adjusted in such a manner to that their hydrophobic and aromatic amino acid residues became more accessible to the polymer surface but their secondary structures remain stable.

### 3.4 Interaction of SARS-CoV-2 S Protein with Different Surfaces: A Comparison of MD Simulation Results

In addition to polystyrene, in our previous publications, we have also investigated the interaction of the S protein with a variety of other hard (graphite and cellulose) and soft (different skin models) materials.^22,23^ In previous works, we considered common materials such as cellulose and graphite and also models of human skin (Sebum, Stratum corneum, and POPC). Hence, it could be interesting to compile together our present and previous results to develop a general scheme or classification summarizing how different materials interact with the SARS-CoV-2 S protein. The results for all of the studied systems are collected in Table 2.

**Table 2:** Comparison of Results for MD Simulations of the Interaction of the SARS-CoV-2 S Protein and Different Surfaces^b^

	cellulose	graphite	sebum^a^	stratum corneum^a^	POPC^a^	polystyrene
number of contacts	51 ± 2	96 ± 2	87 ± 6	180 ± 6	158 ± 8	61 ± 3
number of H-bonds	18 ± 4		11 ± 3	74 ± 8	75 ± 8	
RMSD/Å	8.1 ± 0.2	18.3 ± 0.3	3.8 ± 0.2	6.8 ± 0.1	5.7 ± 0.2	7.62 ± 0.3
tilt angle/deg	45.3 ± 2.3	76.4 ± 0.7	16.2 ± 0.7	83.0 ± 0.5	82.9 ± 1.5	10.1 ± 0.5

a “Sebum” means a model of the sebaceous outer layer of human skin; “stratum corneum” is the nonsebaceous outer layer of human skin; and “POPC” can be justified as a model for soft matter.

b All of the data correspond to the up conformation of the S protein.

**Figure 9:**
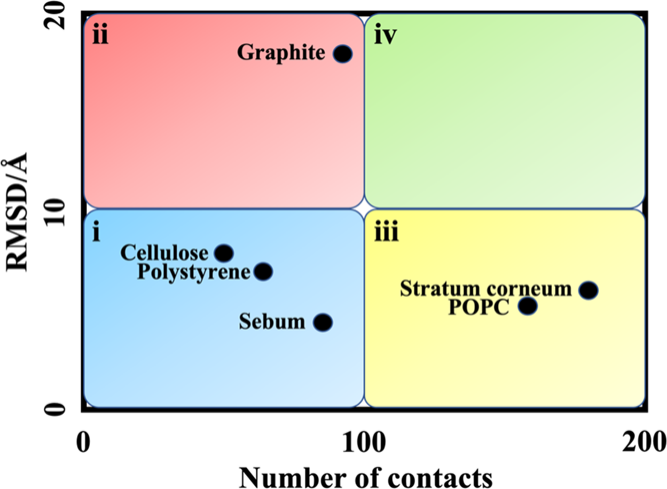
RMSD changes of the SARS-CoV-2 S protein versus total number of contacts during its interaction with different types of materials we have studied using MD simulations. The i–iv classifications are described in the main text and can be used to include also results of future investigations.

From the data compiled in Table 2, we have selected the RMSD and the number of contacts as the quantities to be employed in our classification of the different materials (Figure 9). The RMSD can be considered a representative measure of the structural changes of the protein induced by the interaction with the material, and the total number of contacts measures the affinity of the protein to be adsorbed on the surface of the material. In this regard, we can imagine four different types of materials based on their interaction with SARS-CoV-2 S protein: (i) materials with low affinity for the S protein that are not able to change the protein structure (RMSD and number of contacts are below 10 Å and 100, respectively); (ii) materials with low affinity for the S protein that have high ability to change the protein structure (RMSD above 10 Å and number of contacts below 100); (iii) materials with high affinity for the S protein that are not able to change the protein structure (RMSD below 10 Å and number of contacts above 100); and (iv) materials with high affinity for the S protein that have high ability to change the protein structure, as well (RMSD and number of contacts above 10 Å and 100, respectively). Indeed, group i can be considered as materials that have no special effect on infective viral particles (cellulose, sebum, and polystyrene in our studies); group ii can be considered as materials that may inactivate SARS-CoV-2 virus but are less likely to accumulate infective viral particles (graphite in our studies); group iii are materials with the ability to capture and accumulate the infective viral particles but cannot to inactivate them (POPC and stratum corneum in our studies). Hence, it can be stated that the materials in group iii are able to inhibit transmission. Finally, group iv contains materials that not only have a high affinity for capturing the SARS-CoV-2 virus but also may denature the S protein and inactivate the virus. Materials classified in this group may have the possibility to be used as virucidal materials or main components of personal protective equipment.^48,49^ The presented scheme in Figure 9 can be used to classify the available and future results for the interaction of SARS-CoV-2 S protein with different materials in a reasonable manner.

## 4 Conclusions

A characteristic feature of the SARS-CoV-2 virus is, as in all coronavirus, the presence of a large glycoprotein spike (S) protruding from the viral envelope, which is responsible for the interactions of the virus with the environment and the host. In the present work, we have investigated the interaction of the most external part of the S protein (S1 subunit) of the SARS-CoV-2 virus with a polystyrene surface using all-atom MD simulations. Our study was motivated by the fact that AFM measurements revealed an adhesion force between S and polystyrene stronger than that observed in many other materials, including glass, textiles, and metals. This is a highly relevant fact not only from the point of view of fundamental science (our understanding of protein–surface interactions) but also from the practical point of view since polystyrene is a widely employed material.

In our simulations, we have considered both possible S conformations (up and down) since they are equally present in SARS-CoV-2 virions. We have obtained that the up conformation of S has a stronger interaction with polystyrene (higher interaction energy and higher contact surface) than the S protein in the down conformation. This difference is due to the fact that each S conformation undergoes a different mechanism to be adsorbed on the surface of polystyrene. The main driving forces for adsorption of S in the up conformation were hydrophobic and π–π interactions of polystyrene with the hydrophobic and aromatic residues of the protein, mostly with the amino acids at the exposed RBD and also with the glycans. In the case of the down conformation, all three RBDs of the S protein were hidden and the adsorption was dominated by the interaction between glycans and the polystyrene surface. It is important to recall the important role played by glycans in the interaction of S with surfaces. As pointed out previously,^50^ the high presence of glycans over S plays an essential role by shielding the S protein from the host immune response. As a side effect of this glycan shielding, we observe a strong interaction of S with the polystyrene surface, which is increased in the up conformation due to the role of the exposed RBD.

The estimated adhesion energy per unit surface is similar in both cases (the largest interaction energy for the up case is mostly due to a higher contact area), and it is compatible with the adhesion energy estimated from AFM experiments.

On the other hand, investigation of RMSD, SASA, volume, *R*_g_, and secondary structure of the protein conformations revealed that adhesion to the polystyrene surface did not cause any tangible tertiary or secondary structural changes in the protein conformations. However, the conformational changes for the up conformation were more than those for the down conformation. Evaluation of the protein residues mobility (RMSF) showed that amino acid residues located at RBD with up conformation (Arg319-Phe541) can adjust their conformation to start the interaction with the polymer surface and have an obvious higher RMSF than similar amino acid residues in down conformation. Overall, our results suggest that SARS-CoV-2 virions may adsorb strongly over polystyrene surfaces without significantly affecting (decreasing) its infectivity, suggesting that cleaning/disinfection of highly touched plastic surfaces is recommended.

Finally, we have compiled our previous MD results of the interaction of S with different materials together with our new results obtained for polystyrene in a chart summarizing the affinity of different materials for the SARS-CoV-2 S protein and their ability to change its conformation. Based on our classification, polymeric materials like cellulose and polystyrene have no special effect on infective viral particles, but carbon-based materials like graphite may inactivate SARS-CoV-2 virus. Also, POPC and stratum corneum have the ability to capture and accumulate the infective viral particles but cannot inactivate them.

Our results can shed the light to investigate the fundamental physicochemical aspects of the virus–polymer interaction to identify which factors may make a polymer prone to virus adhesion or make its surface virucidal. Furthermore, the presented classification provides a general view that might pave the way for future studies about the interaction of enveloped viruses with the surface of materials.

## Supporting Information Available

Fully glycosylated structures of the S1 subunit of SARS-CoV-2 spike protein, chemical structure of a styrene monomer, atom types used in CHARMM force field for styrene monomer, RMSD evolution of water droplet, and changes in secondary structure components of the spike protein (PDF)

## Notes

Fully glycosylated structures of the S1 subunit of SARS-CoV-2 spike protein were taken from CHARMM-GUI archive (https://www.charmm-gui.org/?doc=archive&lib=covid19). The polystyrene slab was prepared Maestro-Schrodinger software (https://www.schrodinger.com/products/maestro). Specific open access software from third parties was also used: GROMACS version 2019.3 (https://www.manual.gromacs.org/documentation/2019.3/download.html/) and VMD1.9 (http://www.ks.uiuc.edu/Research/vmd/). Input and output files are available at https://github.com/soft-matter-theory-at-icmab-csic (and also from the corresponding author upon request).
