# Supplementary material for: Molecular Dynamics Simulations of Adsorption of SARS-CoV-2 Spike Protein on Polystyrene Surface
Source: J Chem Inf Model. 2022 Aug 4;62(16):3814–24. doi: 10.1021/acs.jcim.2c00562 (PMC9364975; doi:10.1021/acs.jcim.2c00562)
Supplement: Supplementary file 1 — ci2c00562_si_001.pdf [file ci2c00562_si_001.pdf]

Supporting Information

Molecular Dynamics Simulations of Adsorption of SARS-CoV-2 Spike Protein on Polystyrene Surface

Mehdi Sahihi, Jordi Faraudo*

Institut de Ciencia de Materials de Barcelona (ICMAB-CSIC), Campus de la UAB, E-08193 Bellaterra, Barcelona, Spain

Corresponding author: Mehdi Sahihi; msahihi@icmab.es jfaraudo@icmab.es

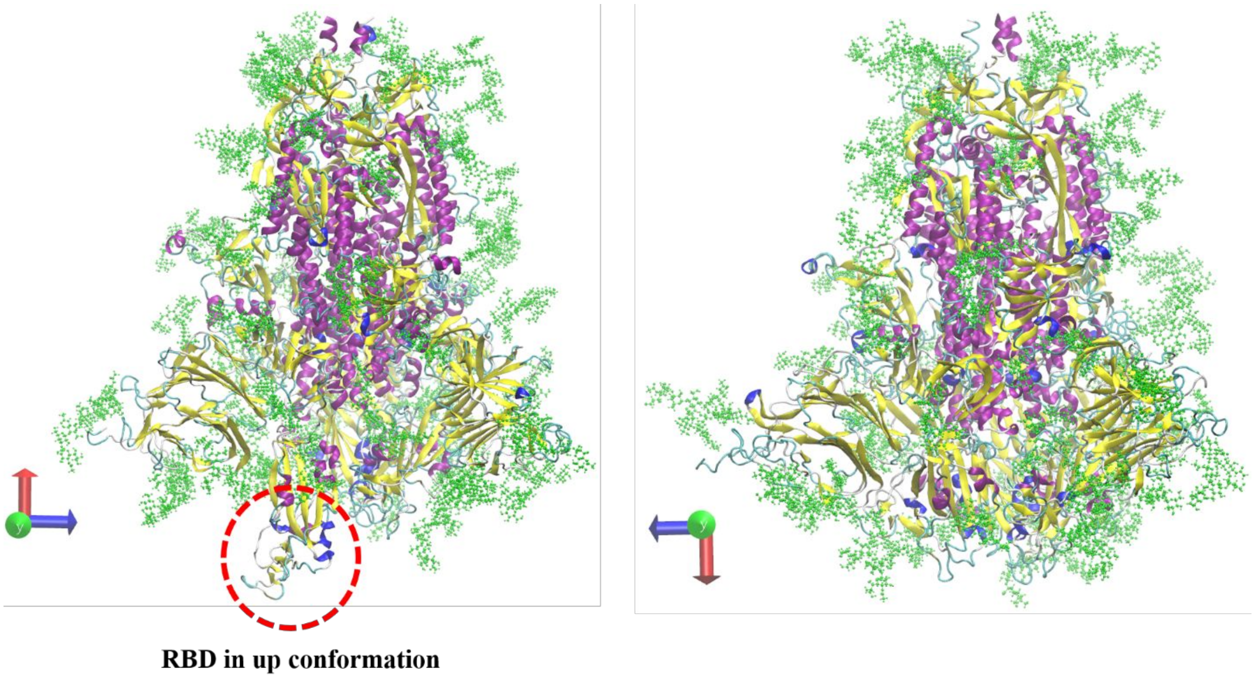

Figure S1. Fully glycosylated structures of the S1 subunit of SARS-CoV-2 spike protein were taken from CHARMM-GUI archive (PDB IDs: 6VSB and 6VXX for up and down conformations, respectively. Glycan groups are shown by green CPK representation.

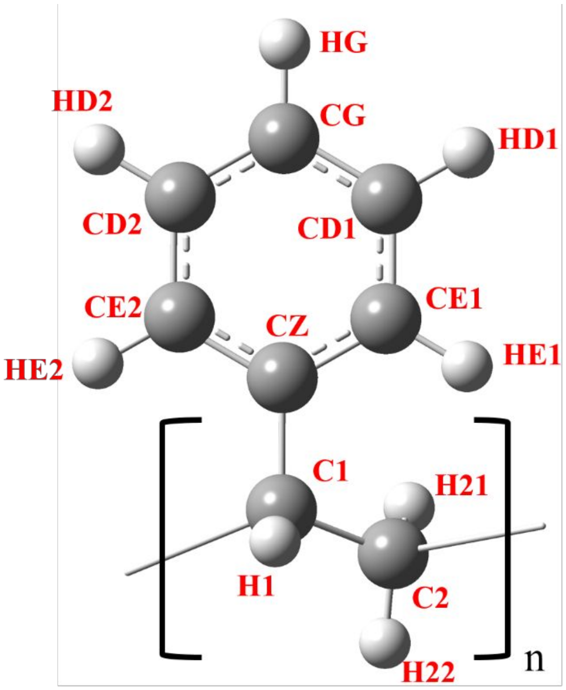

Figure S2. Chemical structure of a styrene monomer with its atomic symbols.

Table S1. Atom types used in CHARMM force field for styrene monomer. (Atomic symbols have been mentioned in **Figure S3**).

Symbol	CHARMM atom type	Element	Atomic charge
CG	CG2R61	C	-0.115
HG	HGR61	H	0.115
CD1	CG2R61	C	-0.115
HD1	HGR61	H	0.115
CD2	CG2R61	C	-0.115
HD2	HGR61	H	0.115
CE1	CG2R61	C	-0.115
HE1	HGR61	H	0.115
CE2	CG2R61	C	-0.115
HE2	HGR61	H	0.115
CZ	CG2R61	C	0.000
C1	CG311	H	-0.090
H1	HGA1	H	0.090
C2	CG321	C	-0.180
H21	HGA2	H	0.090
H22	HGA2	H	0.090

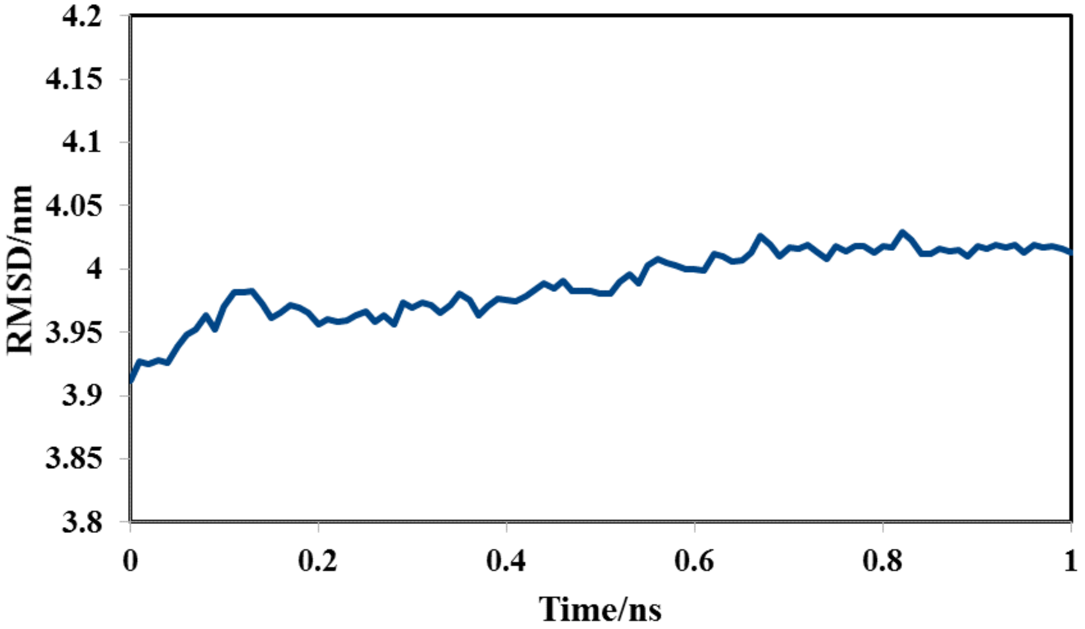

Figure S3. RMSD evolution of water droplet during the interaction with polystyrene surface.

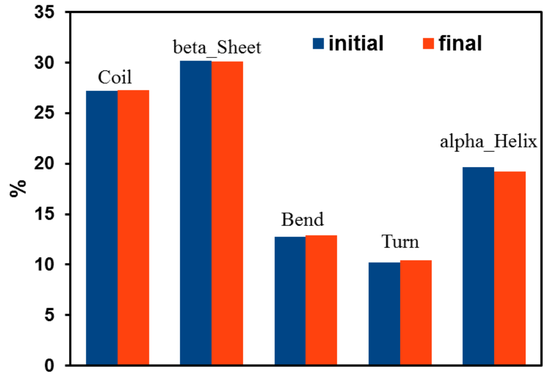

(Up conformation)

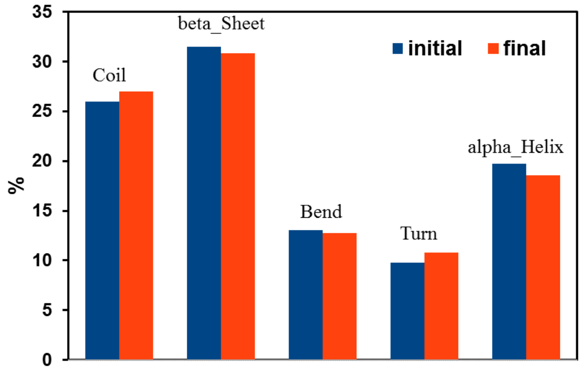

(Down conformation)

Figure S4. Changes in secondary structure components of the spike protein during interaction with polystyrene surface.
